# Right Ventricular and Left Atrial Strain Predict Volumetric Response to Cardiac Resynchronization Therapy

**DOI:** 10.3390/jcdd12040152

**Published:** 2025-04-11

**Authors:** Shing Ching, Jeffrey Ji-Peng Li, Stefanie Maria Werhahn, Rebecca Elisabeth Beyer, Misael Estepa, Christian Stehning, Djawid Hashemi, Natalia Solowjowa, Christoph Klein, Henryk Dreger, Sebastian Kelle, Patrick Doeblin

**Affiliations:** 1 Deutsches Herzzentrum der Charité–Medical Heart Center of Charité and German Heart Institute, Department of Cardiology, Angiology and Intensive Care Medicine, Campus Virchow Klinikum, Augustenburger Platz 1, 13353 Berlin, Germany; csa698@ha.org.hk (S.C.); henryk.dreger@dhzc-charite.de (H.D.);; 2 Charité–Universitätsmedizin Berlin, Corporate Member of Freie Universität Berlin and Humboldt-Universität zu Berlin, Charitéplatz 1, 10117 Berlin, Germany; 3 Division of Cardiology, Department of Medicine and Geriatrics, United Christian Hospital, Hong Kong, China; 4 DZHK (German Centre for Cardiovascular Research), Partner Site Berlin, Potsdamer Str. 58, 10785 Berlin, Germany; 5 Clinical Science, Philips Healthcare, Röntgenstraße 24, 22335 Hamburg, Germany

**Keywords:** cardiac resynchronization therapy, strain, right ventricle, left bundle branch block

## Abstract

Background: While left-bundle-branch-block-related contraction patterns as well as echocardiography-derived strain are variably associated with the volumetric response to cardiac resynchronization therapy (CRT), the role of CMR-derived strain parameters is unexplored. Methods: A total of 50 patients receiving CRT implantation were retrospectively analyzed, all of whom had undergone CMR imaging within one year before, and echocardiography within 6 months before and 6–12 months after CRT implantation. We assessed CMR-derived morphological and functional parameters with regard to the echocardiographic response, defined as a reduction in the left ventricular end-systolic volume of ≥15%. Results: Among the standard CMR parameters, the indexed right ventricular volumes in end-diastole (RVEDVi) (74.5 ± 19.5 vs. 94.8 ± 30.2 mL/m^2^, *p* = 0.006) and end-systole (RVESVi) (43.2 ± 13.3 vs. 61.6 ± 28.8 mL/m^2^, *p* = 0.003), as well as the left atrial (LA) area (24.8 ± 3.5 vs. 30.4 ± 9.5 cm^2^, *p* = 0.020), differed significantly between CRT responders and non-responders. In strain analysis, CRT responders showed a significantly better LA global longitudinal strain (GLS) (25.1 ± 10.4 vs. 15.3 ± 10.5, *p* = 0.002), LA global circumferential strain (GCS) (27.9 ± 14.7 vs. 17.1 ± 13.1%, *p* = 0.012), RV GLS (−25.0 ± 6.5 vs. −18.9 ± 7.6%, *p* = 0.004) and RV free wall strain (−31.1 ± 7.9 vs. −24.9 ± 9.5, *p* = 0.017). Conclusions: CMR-derived peak septal circumferential strain and RVEDVi correlated with the echocardiographic volumetric response to CRT at 6–12 months.

## 1. Introduction

Cardiac resynchronization therapy (CRT) is a cornerstone in the treatment of heart failure with a left bundle branch block (LBBB) and a left ventricular ejection fraction (LVEF) of ≤35% [1]. However, about one-third of patients do not respond favorably, defined as a left ventricular end-diastolic volume (LVESV) reduction of ≥15% at 6–12 months, which is a strong predictor of future adverse events [2]. It was thought that patients with the most mechanical dyssynchrony would derive the maximum benefit from CRT, but the trial results were disappointing [3,4]. Over the years, various parameters have been proposed to predict CRT response, including end-systolic septal strain, systolic stretch index, right ventricular function and myocardial scarring [5], yet most studies were echocardiography-based and examined one parameter at a time in highly selected patient populations. Using the ability of cardiac magnetic resonance (CMR)-based volumetry and feature tracking (FT)-derived strain, we sought to examine the correlation of ventricular size and function, various strain parameters and myocardial scarring with CRT response, defined as an LVESV reduction of ≥15% at 6–12 months after implant, in a heterogenous patient population.

## 2. Materials and Methods

### 2.1. Patient Population

This study retrospectively analyzed patients who underwent CMR scans from January 2012 to January 2022 at our institution within one year prior to successful CRT implantation, who also had a transthoracic echocardiogram within 6 months prior to and 6–12 months after CRT implantation. A total of 50 patients were included for analysis. Clinical information including age, gender, body height and weight, comorbidities, pre-implant ECG and pacing parameters 6–12 months post-implant were collected. The operation records were reviewed to confirm successful CRT implantation with the LV lead in the posterolateral or anterolateral non-apical position. All LV leads were multipolar. This study was approved by the local ethics committee. Informed consent was waived for this retrospective study.

### 2.2. CMR Acquisition

CMR scans were performed on 1.5T (Achieva, Philips Healthcare, Best, The Netherlands) and 3T (Ingenia, Philips Healthcare, Best, The Netherlands) scanners with multi-channel phased-array receiver coils. A standard retrospectively gated balanced steady-state free precession sequence was employed to acquire cine images at the left ventricular short axis and 2-, 3- and 4-chamber views at a minimum of 25 phases per cardiac cycle. Patients received 0.15 mmol/kg of gadobutrol (Gadovist^®^ 1.0 mmol/mL, Bayer AG, Leverkusen, Germany) in scans performed from 2012 to 2020, and 0.1 mmol/kg of gadobutrol in scans from 2021 to 2022 due to an institutional protocol change. Late gadolinium enhancement imaging was performed using a 3D fat–water-separated inversion-recovery sequence (mDIXON) at an inversion time selected based on a Look-Locker sequence starting 5–10 min post contrast injection, depending on the contrast agent dose.

### 2.3. CMR Analysis

**Figure 1:**
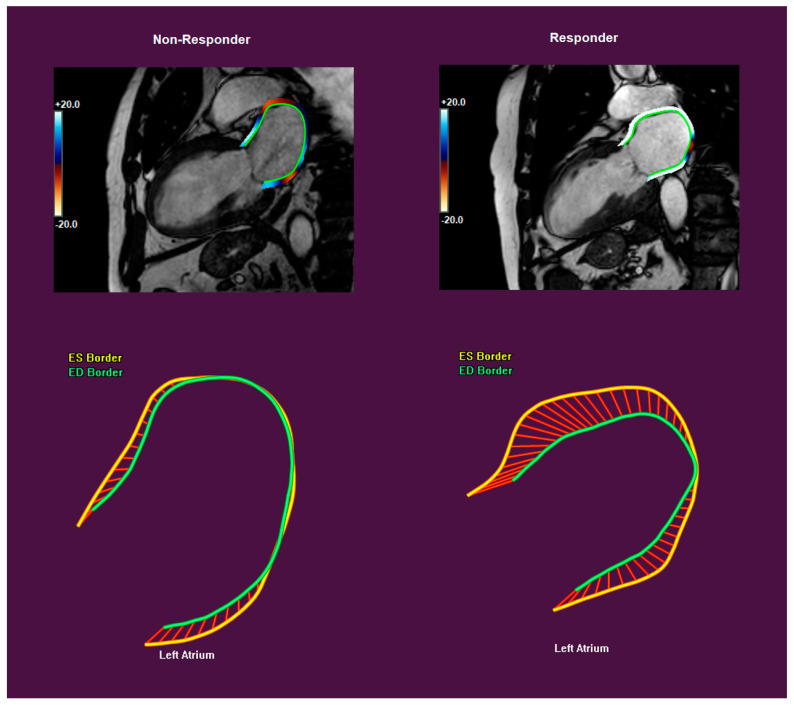
Exemplary contouring for measurements of left-atrial endocardial longitudinal strain in the 2-chamber view (QStrain RE version 4.4, Medis, Leiden, The Netherlands) in a non-responder (**left**) and responder (**right**).

Left- (LV) and right-ventricular (RV) volumetry were performed offline using commercially available postprocessing software (Philips Intellispace Portal Version 12.1, Philips Medical Systems Nederland BV, Best, The Netherlands). LV short-axis end-diastolic and end-systolic cine images were manually contoured to obtain the LV and RV end-diastolic and end-systolic volumes, from which the stroke volume and ejection fraction were calculated. LV and RV volumes were indexed to the body surface area. The left atrial (LA) and right atrial (RA) areas were calculated from end-systolic cine 4-chamber views. A septal scar was defined as any LGE in the basal or mid-ventricular anteroseptal or inferoseptal segments; a lateral scar was similarly defined in the basal or mid-ventricular anterolateral or inferolateral segments. Subendocardial or transmural late gadolinium enhancement (LGE) was classified as ischemic, whereas intramyocardial or epicardial LGE was classified as non-ischemic. Strain analysis was performed using dedicated postprocessing software (QStrain RE version 4.4, Medis, Leiden, The Netherlands). For endocardial strain analysis, LV two-, three- and four-chamber long-axis cine images, as well as LV basal, mid-ventricular and apical short-axis cine images, were contoured and checked for correct tracking to obtain the LV global longitudinal strain (GLS), global circumferential strain (GCS) and segmental peak and end-systolic strain (16-segment model). Exemplary strain measurements in the 4-chamber view are shown in Figure 1. For the RV, GLS, as well as the free wall LS, was analyzed on four-chamber cine images. LA GLS was analyzed bi-plane on four- and two-chamber cine images.

### 2.4. Echocardiographic Analysis and Definition of CRT Response

Pre- and post-implant transthoracic echocardiograms were analyzed on commercially available software (IntelliSpace Cardiovascular 4.1, Philips Medical systems Nederland BV, Best, The Netherlands) using apical two- and four-chamber views for the bi-plane LV end-diastolic and end-systolic volumes and ejection fraction. CRT responders were defined as having a ≥15% reduction in LV end-systolic volume post CRT implant [2].

### 2.5. Statistical Analysis

Continuous variables are expressed as the mean ± standard deviation (SD), and categorical variables are expressed as numbers and percentages. Continuous variables were compared by an independent samples Student’s *t*-test or non-parametric tests in the case of non-normal distribution. The equality of variances was assessed using Levene’s test. Categorical variables were compared between CRT responders and non-responders using a χ^2^ test, or Fisher’s exact test, when any cell had an expected count of less than 5. Data were analyzed with SPSS (version 26, Statistical Package for the Social Sciences, International Business Machines, Inc., Armonk, NY, USA) and R (version 4.4.1, R Foundation for Statistical Computing, Vienna, Austria). Two-sided *p*-values < 0.05 were considered statistically significant.

## 3. Results

Fifty subjects with a mix of ischemic and non-ischemic cardiomyopathy were included. The average age was 68.1 ± 10.5 years and 66% were male, 52% had a history of atrial fibrillation and 22% had valve surgery. The full baseline characteristics, divided into CRT responders and non-responders, are shown in Table 1. The overall response rate was 64%. No correlation was seen with comorbidities including hypertension, diabetes, chronic kidney disease, atrial fibrillation or a history of valve surgery.

### 3.1. Basic CMR Parameters and CRT Response

Basic anatomical and functional CMR parameters are shown in Table 2 and late gadolinium enhancement (LGE) findings in Table 3. One person did not receive contrast agent and was excluded from LGE analysis. Patients with CRT response showed a significantly lower right ventricular end-diastolic volume index (RVEDVi, 74.5 ± 19.5 vs. 94.8 ± 30.2 mL/m^2^, *p* = 0.006), right ventricular end-systolic volume index (RVESVi, 43.2 ± 13.3 vs. 61.6 ± 28.8 mL/m^2^, *p* = 0.003) and LA area (24.8 ± 3.5 vs. 30.4 ± 9.5 cm^2^). In contrast, the left ventricular end-diastolic volume index (LVEDVi), LV GLS, LV GCS and parameters associated with mechanical dyssynchrony in LBBB such as apical rocking and septal flash did not correlate with CRT response, nor did the presence, etiology or location of scarring.

### 3.2. Strain Measurements and CRT Response

**Figure 2:**
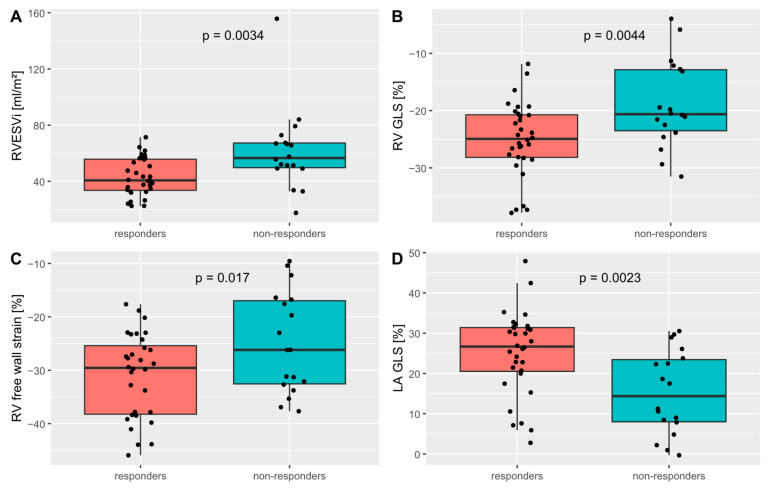
Boxplots of (**A**) right ventricular end-systolic volume index (RVESVi); (**B**) right ventricular global longitudinal strain (RV GLS); (**C**) RV free wall strain; (**D**) left atrial global longitudinal strain (LA GLS) for cardiac resynchronization therapy responders and non-responders. Created using R version 4.4.1. Code available in the Supplementary Materials.

**Figure 3:**
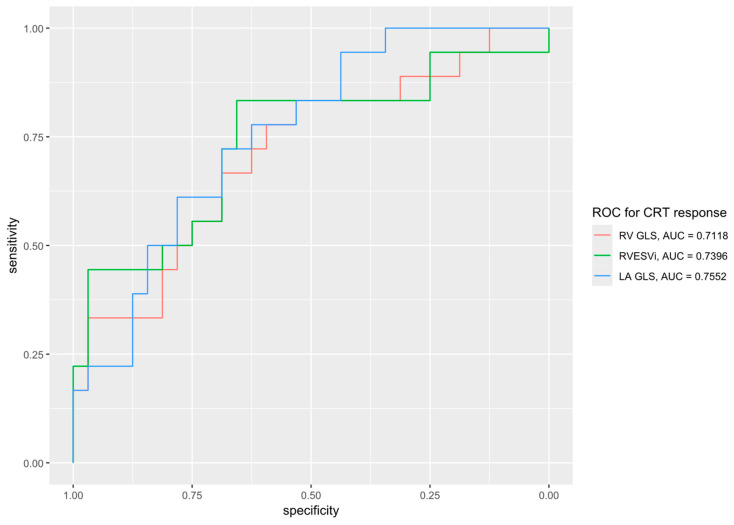
Receiver-operator characteristic (ROC) plot for response to cardiac resynchronization therapy (CRT), defined as a ≥15% reduction in the left-ventricular end-systolic volume post CRT implant, for the predictor variables of right ventricular global longitudinal strain (RV GLS), right ventricular end-systolic volume index (RVESVi) and left atrial global longitudinal strain (LA GLS).

Short- and long-axis strain measurements of responders and non-responders are shown in Table 4 and Table 5. CRT responders showed a significantly better LA global longitudinal strain (GLS) (25.1 ± 10.4 vs. 15.3 ± 10.5, *p* = 0.002), LA global circumferential strain (GCS) (27.9 ± 14.7 vs. 17.1 ± 13.1%, *p* = 0.012), RV GLS (−25.0 ± 6.5 vs. −18.9 ± 7.6%, *p* = 0.004) and RV free wall strain (−31.1 ± 7.9 vs. −24.9 ± 9.5). Other strain parameters were not associated with CRT response. Importantly, the peak and end-systolic septal and lateral LS and CS did not correlate with CRT response. Scatter plots of the RVEDVi and peak septal circumferential strain versus LVESV change at 6–12 months are shown in Figure 2. A receiver-operator characteristic (ROC) plot for RV GLS, RVESVi and LA GLS is depicted in Figure 3.

## 4. Discussion

This study examined the correlation of a comprehensive panel of CMR parameters with CRT response in a patient population that reflects the real-world clinical complexity. For example, 52% had atrial fibrillation and 22% had a history of valve surgery. The overall response rate of 64% in this study is on par with that reported elsewhere [3]. To our knowledge, this is the first study to show the discriminatory value of right ventricular and left atrial strain on CRT response against parameters including scar etiology and location, volumetry and a comprehensive list of LV strain parameters. The main findings are as follows:

(1) RV volumes and strain measurements correlated with CRT response;
(2) LA area and strain measurements correlated with CRT response;
(3) Left ventricular strain parameters and the presence and location of scarring did not correlate with CRT response.

### 4.1. LV Strain and CRT Response

In contrast to previous reports, none of the left ventricular strain parameters examined correlated with CRT response [6,7]. Differences in the study population, study methodology and postprocessing software could be accountable and further research is necessary to further assess the potential discriminatory value of left ventricular strain measurements.

### 4.2. The RV and CRT Response

Previous reports on the RV and CRT response are mixed. A metanalysis of 17 studies with echocardiography-derived RV functional parameters found no correlation with CRT response [8] for any of the examined parameters. Studies using CMR, which is considered the gold standard for right ventricular measurements, found mixed results. In two studies, RVEF and tricuspid annular plane systolic excursion (TAPSE) in CMR were reported to correlate with CRT response [9,10]. Another study found no correlation between RV function in CMR and mortality after CRT but did not report CRT response and had a highly selected patient population with a mean RVEDVi of only 64 mL/m^2^, which is much lower than that in our cohort [11]. Additionally, none of the aforementioned CMR studies examined RV strain, less than 20% of subjects had atrial fibrillation and only 1% of subjects had undergone valvular surgery.

### 4.3. The LA and CRT Response

Consistent with previous reports [12,13,14], we showed that larger LA size and impaired LA GLS and GCS were associated with CRT non-response. The presence of atrial fibrillation could not be accountable, since its incidence as well as the biventricular pacing percentage was similar in responders and non-responders (Table 5). Although LA dysfunction may signify more advanced LV disease, there was no significant difference in LVEDVi, LV GLS or LV GCS. The mechanism linking LA function and CRT response remains unclear and should be the focus of further studies.

### 4.4. Scar and CRT Response

Previous studies reported lower rates of CRT response in the presence of septal scarring [15,16]. This finding could not be replicated in our study, which may be due to the small sample size. Similarly, we could not reproduce the adverse effect of ischemic cardiomyopathy on post-CRT reverse remodeling.

Lateral scarring on CMR has been associated with worse lateral circumferential strain and CRT non-response for concordantly placed LV leads [17], which again could not be reproduced in our study. Among our subjects with lateral scarring, all but one had an LGE ≤50% wall thickness. It is plausible that more extensive (>50% wall thickness) lateral scarring would negatively impact CRT response.

### 4.5. Limitations

A limitation of this study is the small sample size, prohibiting meaningful subgroup analyses. As no Bonferroni correction was performed for the large number of variables tested, all results should be considered as hypothesis-generating. Not all patient characteristics were profiled, including their list of medications, blood pressure, smoking status and history of chemotherapy. Our study is based on strain analysis by one of several commercially available postprocessing software programs (QStrain RE version 4.4, Medis, Leiden, The Netherlands). Whether the results are reproducible on other platforms is unclear, as strain measurements have been shown to vary depending on the postprocessing software used [18].

## 5. Conclusions

In our hypothesis-generating retrospective analysis, CMR-derived strain measurements of the LA and RV, but not the LV, correlated with CRT response, defined as an LVESV reduction of ≥15% at 6–12 months.

This article is a revised and expanded version of a paper entitled ‘Right ventricular and left atrial strain predict volumetric response to cardiac resynchronization therapy’, which was presented at the CMR 2025 congress in Washington on 31 January 2025 [19].

## Figures and Tables

**Table 1:** Baseline parameters of CRT responders and non-responders.

Parameter	Sample Size (*n*)	All Subjects	Responders (*n* = 32)	Non-Responders (*n* = 18)	*p*-Value
Age (years)	50	68.1 ± 10.5	67.7 ± 11.4	68.9 ± 9.0	0.71
Male gender (*n*)	50	33 (66%)	19/32 (59%)	14/18 (78%)	0.23
BMI (kg/m^2^)	50	27.0 ± 4.3	26.4 ± 3.9	28.0 ± 4.9	0.19
Hypertension (*n*)	50	29 (58%)	19/32 (59%)	10/18 (56%)	>0.99
Diabetes mellitus (*n*)	50	14 (28%)	8/32 (25%)	6/18 (33%)	0.53
Chronic kidney disease (*n*)	50	23 (46%)	12/32 (38%)	11/18 (61%)	0.14
CAD (*n*)	50	25 (50%)	14/32 (43.8%)	11/18 (61.1%)	0.24
Past chemotherapy (*n*)	50	5 (10%)	1/32 (3.1%)	4/18 (22.2%)	0.05
History of smoking (*n*)	50	5 (10%)	3/32 (9.4%)	2/18 (11.1%)	>0.99
Atrial fibrillation (*n*)	50	26 (52%)	15/32 (47%)	11/18 (61%)	0.39
Valve surgery (*n*)	50	11 (22%)	5/32 (16%)	6/18 (33%)	0.17
Betablocker (*n*)	50	48 (96%)	30/32 (93.8%)	18/18 (100%)	0.53
ACE-inhibitor (*n*)	50	15 (30%)	9/32 (28.1%)	6/18 (33.3%)	0.70
AT1-antagonist (*n*)	50	9 (18%)	6/32 (18.8%)	3/18 (16.7%)	>0.99
ARNI (*n*)	50	23 (46%)	15/32 (46.9%)	8/18 (44.4%)	0.87
SGLT2-inhibitor (*n*)	50	5 (10%)	5/32 (15.6%)	0/18 (0%)	0.15
Aldosterone-antagonist (*n*)	50	30 (60%)	20/32 (62.5%)	10 (33.3%)	0.63
Ivabradine (*n*)	50	5 (10%)	3/32 (9.4%)	2/18 (11.1%)	>0.99
Heart rate (/min)	50	72.6 ± 14.1	73.8 ± 15.4	70.4 ± 11.5	0.42
BP systolic (mmHg)	50	125.3 ± 20.9	129.2 ± 22.1	118.3 ± 16.9	0.08
BP diastolic (mmHg)	50	71.5 ± 11.6	71.6 ± 10.4	71.3 ± 13.8	0.92
NYHA II (*n*)	48	30 (62.5%)	22/31 (71%)	8/17 (47%)	0.13
NYHA III (*n*)	48	18 (36%)	9/31 (29%)	9/17 (53%)	
LBBB (*n*)	50	41 (82%)	26/32 (81%)	15/18 (83%)	>0.99
QRS duration (ms)	50	150.3 ± 27.6	150.3 ± 27.5	150.3 ± 28.6	>0.99
Biventricular pacing (%)	44	97.1 ± 3.8	97.5 ± 3.4	96.5 ± 4.6	0.72 ^†^
Baseline echo LVEDV (mL)	50	202.6 ± 66.9	200 ± 65.9	207.3 ± 70.3	0.71
Baseline echo LVESV (mL)	50	142.4 ± 59.0	140.7 ± 57.9	145.4 ± 62.5	0.79
Baseline echo LVEF (%)	50	31.0 ± 11.4	30.5 ± 12.2	31.9 ± 10.1	0.69
LVEDV change (%)	50	−16.9 ± 26.1	−31.6 ± 17.8	9.3 ± 15.5	<0.001
LVESV change (%)	50	−19.1 ± 35.8	−40.5 ± 17.5	19.0 ± 27.1	<0.001

Data are given as the mean ± SD or *n* (%). BMI, body mass index; BP, blood pressure; CAD, coronary artery disease; LBBB, left bundle branch block; LV, left ventricular; LVEDV, LV end-diastolic volume; LVEF, LV ejection fraction; LVESV, LV end-systolic volume; NYHA, New York Heart Association. ^†^ non-parametric test due to non-normal distribution (per Kolmogorov–Smirnov test).

**Table 2:** Standard CMR parameters of CRT responders and non-responders.

Parameter	Sample Size (*n*)	All Subjects	Responders (*n* = 32)	Non-Responders (*n* = 18)	*p*-Value
LVEDd (mm)	50	64.3 ± 8.7	63.1 ± 9	66.4 ± 7.8	0.18
Septal thickness (mm)	50	12.3 ± 3.1	12.7 ± 2.9	11.6 ± 3.3	0.11
Posterior thickness (mm)	50	7.7 ± 1.6	7.8 ± 1.5	7.6 ± 1.7	0.19
LVEDVi (mL/m^2^)	50	122.5 ± 37.7	115.7 ± 37.1	134.7 ± 36.5	0.06
LVEF (%)	50	32.9 ± 9.6	33.6 ± 9.5	31.7 ± 9.9	0.69
RVEDVi (mL/m^2^)	50	81.8 ± 25.6	74.5 ± 19.5	94.8 ± 30.2	0.006
RVESVi (mL/m^2^)	50	49.8 ± 21.9	43.2 ± 13.3	61.6 ± 28.8	0.003
RVEF (%)	50	40.0 ± 11.8	41.8 ± 11.1	36.9 ± 12.5	0.17
LA area (cm^2^)	50	26.8 ± 8.3	24.8 ± 6.8	30.4 ± 9.5	0.020
RA area (cm^2^)	50	23.9 ± 8.0	22.5 ± 5.9	26.4 ± 10.6	0.10
Septal flash (*n*)	49	35 (71.4%)	23/31 (74%)	12/18 (67%)	0.74
Apical rocking (*n*)	49	31 (63.3%)	18/31 (58%)	13/18 (72%)	0.37

Data are given as the mean ± SD or *n* (%). ECV, extracellular volume; LA, left atrium; LV, left ventricular; LVEDd, LV end-diastolic diameter; LVEDVi, LV end-diastolic volume index; RA, right atrium; RV, right ventricular; RVEDVi, RV end-diastolic volume index; RVEF, RV ejection fraction; RVESVi, RV end-systolic volume index.

**Table 3:** Scarring on late gadolinium enhancement imaging in CRT responders and non-responders.

Parameter	All Subjects (*n* = 49)	Responders (*n* = 32)	Non-Responders (*n* = 17)	*p*-Value
Any scar (*n*)	27 (55%)	17 (53%)	10 (59%)	0.77
Ischemic scar (*n*)	15 (31%)	8 (25%)	7 (41%)	0.33
Septal scar (*n*)	13 (27%)	9 (28%)	4 (24%)	>0.99
Lateral scar (*n*)	10 (20%)	5 (16%)	5 (29%)	0.29

**Table 4:** CMR short-axis strain measurements of CRT responders and non-responders.

Parameter	All Subjects (*n* = 50)	Responders (*n* = 32)	Non-Responders (*n* = 18)	*p*-Value
LV GCS (%)	−14.5 ± 6.6	−14.9 ± 6.0	−13.8 ± 6.0	0.56
Peak septal CS (%)	−14.9 ± 8.9	−16.0 ± 8.8	−12.9 ± 9.0	0.23
Peak lateral CS (%)	−22.3 ± 10.5	−23.9 ± 11.5	−19.5 ± 7.9	0.16
End-systolic septal CS (%)	−10.3 ± 10.2	−11.3 ± 9.6	−8.5 ± 11.3	0.37
End-systolic lateral CS (%)	−19.3 ± 10.1	−20.0 ± 11.3	−17.9 ± 7.5	0.48

Data are given as the mean ± SD or *n* (%). CS, circumferential strain; GCS, global circumferential strain; LA, left atrium; LV, left ventricular; RV, right ventricular. One subject was excluded from strain analysis due to insufficient image quality.

**Table 5:** CMR long-axis strain measurements of CRT responders and non-responders.

Parameter	All Subjects (*n* = 50)	Responders (*n* = 32)	Non-Responders (*n* = 18)	*p*-Value
LV GLS (%)	−10.4 ± 3.9	−11.1 ± 3.8	−9.2 ± 4.0	0.10
Peak septal LS (%)	−10.7 ± 5.1	−10.9 ± 4.6	−10.3 ± 6.2	0.67
Peak lateral LS (%)	−24.8 ± 9.4	−26.1 ± 10.1	−22.4 ± 7.5	0.18
End-systolic septal LS (%)	−6.2 ± 7.7	−6.4 ± 7.8	−5.9 ± 7.7	0.82
End-systolic lateral LS (%)	−21.2 ± 11.4	−22.2 ± 13.2	−19.3 ± 7.0	0.38
RV GLS (%)	−22.8 ± 7.4	−25.0 ± 6.5	−18.9 ± 7.6	0.004
RV free wall GLS (%)	−28.9 ± 8.9	−31.1 ± 7.9	−24.9 ± 9.5	0.017
LA GLS (%)	21.6 ± 11.3	25.1 ± 10.4	15.6 ± 10.4	0.002
LA GCS (%)	24.0 ± 15.0	27.9 ± 14.7	17.1 ± 13.1	0.012

Data are given as the mean ± SD or *n* (%). GLS, global longitudinal strain; LA, left atrium; LS, longitudinal strain; LV, left ventricular; RV, right ventricular. One subject was excluded from strain analysis due to insufficient image quality. One subject was excluded from longitudinal strain analysis due to incorrect triggering in the long-axis images.

## Data Availability

Data are available from the corresponding author upon reasonable request.

## Supplementary Materials

The following supporting information can be downloaded at: https://www.mdpi.com/article/10.3390/jcdd12040152/s1, the R-scripts used for the statistical analysis.

## Abbreviations

The following abbreviations are used in this manuscript:

CMR	Cardiac magnetic resonance
CRT	Cardiac resynchronization therapy
CS	Circumferential strain
ECV	Extracellular volume
FT	Feature tracking
GCS	Global circumferential strain
GLS	Global longitudinal strain
LA	Left atrium
LBBB	Left bundle branch block
LGE	Late gadolinium enhancement
LS	Longitudinal strain
LV	Left ventricle
LVEDd	Left ventricular end-diastolic diameter
LVEDV	Left ventricular end-diastolic volume
LVEDVi	Left ventricular end-diastolic volume index
LVEF	Left ventricular ejection fraction
LVESV	Left ventricular end-systolic volume
NYHA	New York Heart Association
RA	Right atrium
ROC	Receiver-operator characteristic
RV	Right ventricle
RVEDVi	Right ventricular end-diastolic volume index
RVEF	Right ventricular ejection fraction
RVESVi	Right ventricular end-systolic volume index
SD	Standard deviation
T	Tesla
TAPSE	Tricuspid annular plane systolic excursion
